# LONGEVITY IN CULTURAL CONTEXT: FINDINGS FROM FOUR CENTENARIAN STUDIES

**DOI:** 10.1093/geroni/igae098.0380

**Published:** 2024-12-31

**Authors:** Peter Martin, Leonard Poon

**Affiliations:** Chair; Discussant

## Abstract

Life expectancy varies across cultural and international groups, but each culture contains long-lived individuals. To what extent survivors in different cultural groups have common or divergent characteristics is still being determined. The purpose of the symposium is to compare and contrast physical, functional, and mental health characteristics among four different cultural groups of centenarians: African American centenarians from the Health and Retirement Study, Hawaii centenarians of Japanese ancestry from the Kuakini Hawaii Centenarian Study, Japanese centenarians, and Native Americans from the Oklahoma Centenarian Study. The first study showcases longevity predictors for African Americans in the Health and Retirement Study, highlighting family longevity, individual and family resources, health behaviors, and physical, functional, and mental health. The second study utilizes centenarian data from the Kuakini Hawaii Centenarian Study, featuring their unique survivorship characteristics as participants age from 70 to 100 years. The third presentation highlights Japanese centenarians and reveals the diversity of physical and mental well-being of centenarians in Japan. Findings highlight the high bed-bound ratio among centenarians in Japan. Finally, the Oklahoma Centenarian Study identifies themes pertaining to underlying cultural influences using oral narratives of centenarian descendants of indigenous ancestry. The themes of “transmission of historical trauma,” “shared collectivism,” “leisure and sporting,” and “educational attainment“signify themes of adaptation and survival. The four centenarian studies suggest similarities and essential differences when comparing these exceptional cultural groups of survivors. Practitioners and policymakers should be responsive to the diverse needs of centenarians who provide cultural individuality to their communities.

